# Characterization of a Low Pathogenic Avian Influenza Virus (H6N1) Isolated from Turkeys

**DOI:** 10.1155/2011/285218

**Published:** 2010-12-30

**Authors:** I. Shkoda, E. Lapin, E. Rosenbluth, S. Perk, Z. Geva, A. Inbar, I. Davidson

**Affiliations:** ^1^Division of Avian Diseases, Kimron Veterinary Institute, P.O. Box 12, Bet Dagan 50250, Israel; ^2^Yeffe-Hod, Turkey Breeding Farms and Hatcheries, Kvutzat Yavne, P.O Evtach, 79233, Israel; ^3^Israel Veterinary Services, Southern Diagnostic Laboratory for Poultry, Beer Tuvia 79540, Israel

## Abstract

An avian influenza virus (AIV), A/turkey/Israel/09 subtype H6N1, was isolated from turkey poults exhibiting typical pathology associated with AIV infection. The virus was characterized by RT-PCR using AIV subtype-specific primers and by the haemagglutination inhibition test using AIV subtype-specific antisera. The virus has an intravenous pathogenicity index of 0 and possessed a nucleotide sequence at the cleavage site of the hemagglutinin gene, PQIETR*GLF, associated with avian influenza viruses of low pathogenicity. Unlike the two previous H6N2 isolates originating from domestic ducks and mallard, the A/turkey/Israel/09 (H6N1) was isolated from turkeys. The gene sequences of the A/turkey/Israel/09 (H6N1) virus show divergence from the former Israeli H6 isolates.

## 1. Introduction

The influenza viruses include three genera, A, B, and C within the family *Orthomyxoviridae.* Avian influenza viruses (AIVs), all of which are contained in the genus influenza virus A, are an economically important cause of disease in fowl and occasionally affect humans, pigs, and horses [1, 2].

Each genus of the virus is further subdivided into serotypes based on the surface proteins, consisting of 16 different hemagglutinin (H) and 9 neuraminidase (N) subtypes. While only a limited number of H and N subtypes are circulating in humans and other mammalian species, all the H and N subtypes are found in avian species [3, 4]. The genome of influenza A viruses consists of 8 unique segments of single-stranded negative sense RNA. The viral RNA segments encode 10 recognized gene products, PB1, PB2, and PA polymerases, H, NP, N, M1, M2, NS1, and NS2 proteins [5].

The H6 subtype is one of the most commonly recognized subtypes in domestic ducks in southern China [6, 7] and in migratory birds in North America and in Europe [8–10]. H6 viruses have caused several outbreaks in commercial poultry worldwide that resulted in decreased egg production and increased mortality [11–13]. During the Hong Kong H5N1 “bird flu” incident in 1997, an H6N1 avian influenza virus, teal/Hong Kong/W312/97 (W312), was isolated from birds in a live poultry market [14]. Genetic characterization of this virus revealed that except for the H gene, the remaining 7-gene segments were closely related to those of highly pathogenic avian influenza H5N1 viruses (HPAIVs) found in both poultry and humans [14]. 

The present study reports on the outbreak of avian influenza (AI) caused by an H6N1 subtype of AIV isolated during January 2009 in Kibbutz Gvulot, in the southern part of Israel. The present isolate, A/turkey/Israel/09 (H6N1), is the third H6 AIV obtained in Israel since the year 2000. Therefore, circulation of these viruses was concurrent with that of the highly prevalent H9N2 AIV in Israel [15, 16]. While the former H6 AIV isolates, A/duck/Israel/289/01 (H6N2) and A/mallard/Israel/320/01 (H6N2), were isolated from water birds, the present isolate was obtained from domestic turkey poults.

## 2. Materials and Methods

### 2.1. Description of the Flock of Origin

Six week-old turkey poults in one of six poultry houses (no. 2) located in one farm, Kibbutz Gvulot, exhibited an increased daily mortality of approximately 1% over a two-day period. The clinical signs were swollen infraorbital sinuses, nasal discharge, respiratory rales, rattles,and lethargy. Pneumonia, pericarditis, air sacculitis and tracheal discharge were observed on postmortem examination. Birds in the affected poultry house had developed AIV antibodies, as detected by ELISA and AIV H6 haemagglutination inhibition (HI) tests. The ELISA test was performed with the FlockChek Avian Influenza Antibody Test Kit (IDEXX, USA) according to the manufacturer's instructions, and the HI test employed a panel of reference antisera to H5, H6, H7, and H9. Blood samples were tested for antibodies by the HI test using 4 (HA) units of the H6 subtype antigen [17]. Turkey poults from the six houses were distributed to 4 secondary farms, Mishmeret, Ramon, Ein Zurim, and Evron.

### 2.2. Virus Detection

Tracheal and cloacal swabs of clinically-affected turkeys were assayed for the presence of AIV by PCR and virus isolation in 11-day-old embryonated eggs [17]. The presence of AIV was initially detected in the allantoic fluids (AF) of dead eggs after 2-3 days of incubation, by the hemagglutination (HA) assay [17]. Testing of HA-positive AF was performed by reverse transcriptase polymerase chain reaction (RT-PCR). In parallel, RT-PCR was performed on RNA extracted directly from tracheal and cloacal swabs.

### 2.3. Molecular Characterization of AIV

Viral RNA was extracted directly from the tracheal and cloacal swabs and from AF using the QIAamp Viral RNA Mini Kit (Qiagen, Valencia, CA) according to the manufacturer's instructions. The purified RNA was then tested by RT-PCR. 

Firstly, AIV was detected using a primer pair specific for the M gene of influenza A virus, Mf (5′-TTCTAACCGAGGTYRAAACGT-3′) and Mr (5′-CTGGGCACGGTGAGCGT-3′) to amplify a 200 bp product (Panshin A., Kimron Veterinary Institute, Bet Dagan, Israel, unpublished). RT-PCR was performed using a One Step RT-PCR kit, (QIAGEN Ltd.). A 25 *μ*l mixture containing 5 *μ*l of buffer, 1 *μ*l of dNTP mix, 1 *μ*l of each primer, 1 *μ*l of One Step Enzyme mix, 13 *μ*l nuclease-free water, and 3 *μ*l RNA template was used in the assay as follows: 30 min at 50°C, 10 min at 95°C, 30 cycles at 94°C for 30 sec, then 30 sec at 54°C, 30 sec at 72°C, 5 min at 72°C, and 15 min at 4°C using the DNA Engine thermal cycler (Bio Rad, Waltham, MA, USA). The RT-PCR product was separated by electrophoresis in 2% agarose gel.

Secondly, molecular subtyping was performed with a set of specific primers for genes of H subtypes H1–H4, H6, H9, and H10 [18] and H5, H7 [19], and N subtypes N1 [20] N2, N3, N7, N9 (Panshin A., Kimron Veterinary Institute, Bet Dagan, Israel, unpublished). The primers used to amplify a 623 bp segment of the N2 gene were N2f (5′-YTGYACAGTRGTAATGACBGATGGR-3′) and N2r (5′-CRATRCTRYTTGADGTCCACCABAC-3′); The primers to used to to amplify a 470 bp product of the N3 gene were N3f (5′-AAYAGRCCWTGGRTGAGRAT-3′) and N3r (5′-CCCGATCCAGGTTCATTGT-3′). The primers used to amplify a 509 bp segment of the N7 gene were N7f (5′-TTGGRTGGTCRAGYACAAGCTGCC-3′) and N7r (5′-ACCAGGRCTYCCAGTDATWGGATTG-3′), the primers used to amplify a 520 bp segment of N9 gene were N9f (5′-CAACCAATGCAAGCCAAACAATA-3′) and N9r (5′-TTTCGGGCCCATGTGTTGATTTC-3′). The amplification conditions for H and N genes were similar to those of the M gene.

### 2.4. Sequence Analysis

The PCR products were purified with the MEGAquick-spin PCR & Agarose Gel Extraction System (iNtRON Biotechnology, Inc, Gyeonggi-do, Korea) according to the manufacturer's instructions. Sequencing reactions were performed by the Weizmann Institute of Science, Rehovot, Israel, using a 3700 DNA Analyzer (Perkin-Elmer, Applied Biosystems, Foster City, CA., U.S.A.) and analysed by capillary electrophoresis. The BioEdit Package, Version 7.3, and DNASTAR system software were used for sequence analysis and alignment.

### 2.5. Phylogenetic Analysis

Nucleotide sequences of all gene segments and available sequence data from GenBank were used to generate phylogenetic trees. The phylogenetic analysis was performed with the MegAlign programme of the DNASTAR system Software.

### 2.6. IVPI Test

The intravenous pathogenicity index (IVPI) of AIV isolates was determined as described in the OIE Manual of Standards for Diagnostic Tests and Vaccines [21]. The test employs a standard volume of virus which is inoculated intravenously into SPF chickens. The IVPI test yields an index of virulence valued from 0 to 3, which is calculated according to illness severity and the viability period following inoculation [21].

## 3. Results and Discussion

### 3.1. Virus Transmission

The Mishmeret farm received poults from the original poultry houses nos. 1 and 4 and developed a low level of HI antibodies (geometric mean titer (GMT) = 1.8); the Evron farm received poults from the original poultry houses nos. 5, and 6 and did not develop HI antibodies (GMT = 0); the Ramon farm received poults from the original poultry houses nos. 1, 4, 5 and 6, and did not develop HI antibodies (GMT = 0); the Ein Zurim farm received poults from the original poultry house no. 2, that was infected with the A/turkey/Israel/09 (H6N1) isolate, and from house no. 3. As a result, birds on the Ein Zurim farm developed HI antibodies to H6 and showed clinical signs (GMT = 6.3).

### 3.2. Virus Identification and Virulence Determination

To identify the AIV H and N gene, specificity amplifications and HI assays were performed indicating the H6N1 subtype of the isolate. The virus was isolated and characterized by classical methods (OIE) and genome was detected by PCR. A/turkey/Israel/09 (H6N1) was characterized as low pathogenic avian influenza virus (LPAI) with an IVPI of 0.00. Infection in the chickens used in the IVPI assay was confirmed by the HI test.

### 3.3. Genetic Characterization of the Gene Cleavage Site

The amino acid sequence of the H gene cleavage site was examined to support the virulence determination of the A/turkey/Israel/09/(H6N1) isolate. The amino acid sequence at the cleavage site was PQIETR*GLF, indicating that the isolate was of low pathogenicity, since it did not contain multiple basic amino acids, characteristic of HPAIVs. Most LPAIVs have a single arginine at the cleavage site, whereas HPAIVs usually exhibit a multibasic amino acid motif (R and K) flanking the cleavage site [22, 23].

### 3.4. Phylogenetic Analyses of the A/Turkey/Israel/09/(H6N1) Isolate Genes

**Figure 1:**
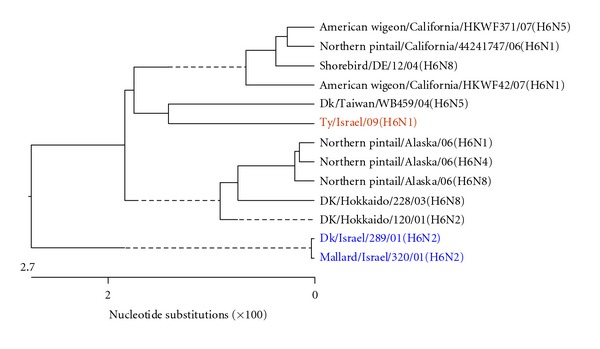
Phylogenetic tree of the H gene of H6 subtype. Here and in the Figures 3–8, the analysis was based on corresponding complete genes.

**Figure 2:**
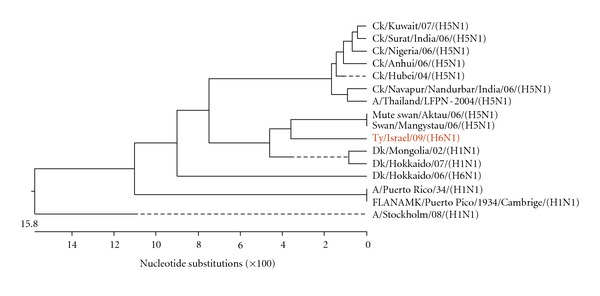
Phylogenetic tree of the N gene of N1 subtype. Analysis was based on nucleotides 204 to 1149 of the N gene.

**Figure 3:**
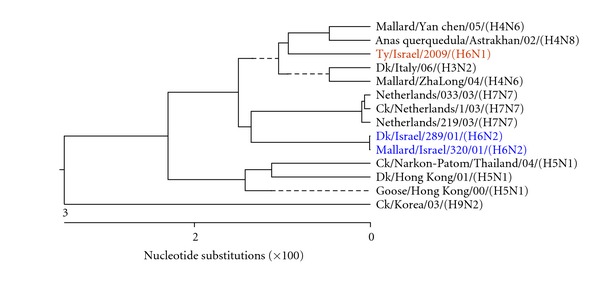
Phylogenetic tree of the NS gene.

**Figure 4:**
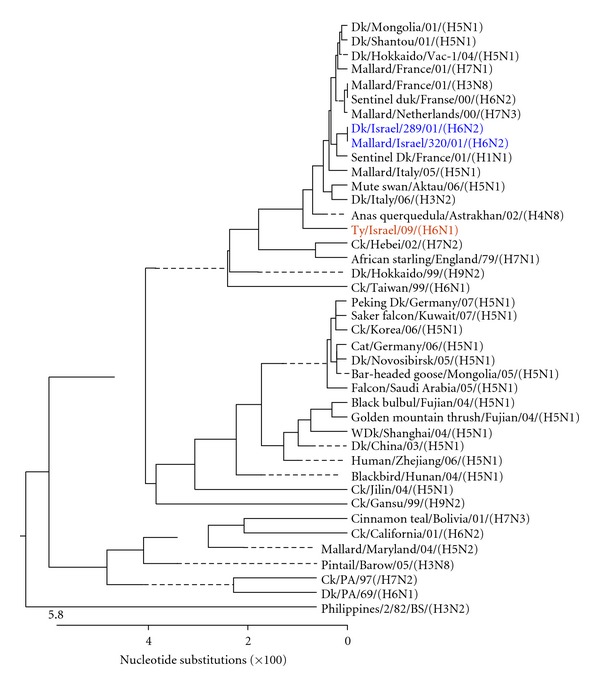
Phylogenetic tree of the M gene.

**Figure 5:**
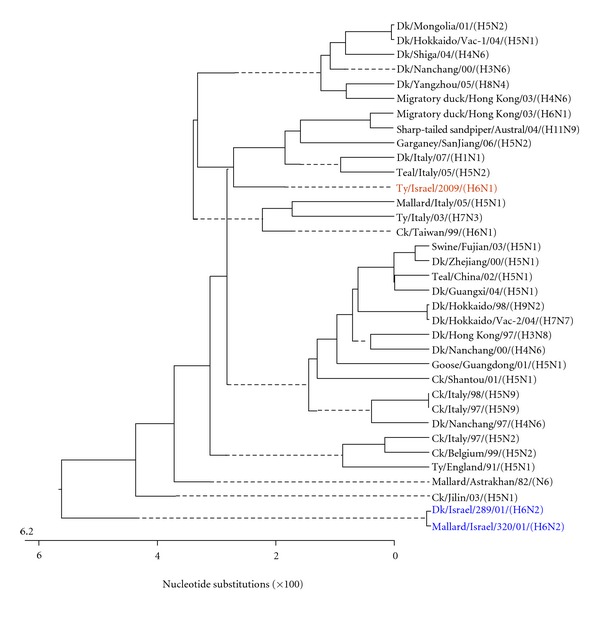
Phylogenetic tree of the NP gene.

**Figure 6:**
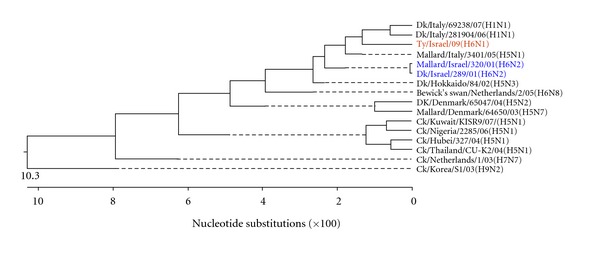
Phylogenetic tree of the PA gene.

**Figure 7:**
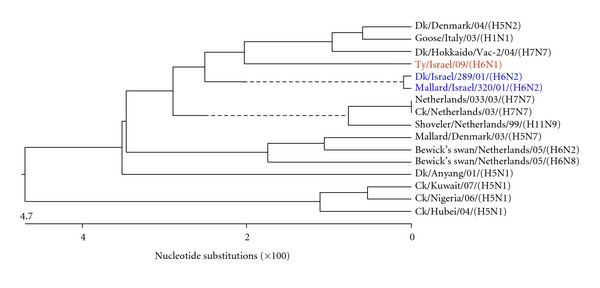
Phylogenetic tree of the PB1 gene.

**Figure 8:**
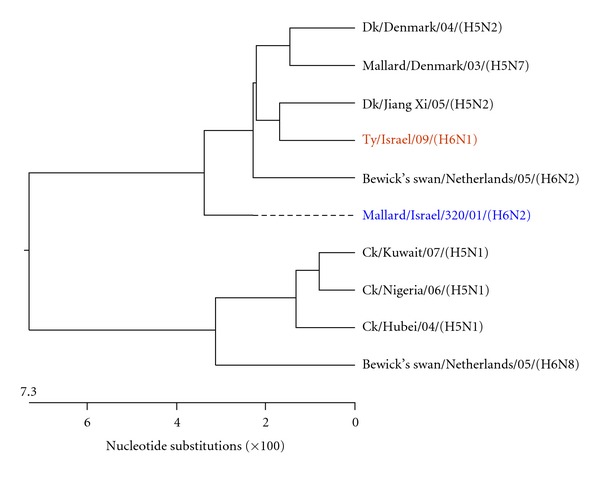
Phylogenetic tree of the PB2 gene.

The genome of influenza A viruses consists of eight unique segments of single-stranded RNA, which are of negative polarity (i.e., complementary to the mRNA sense) [5]. The 8 gene segments of the A/turkey/Israel/09 (H6N1) isolate were sequenced. The nucleotide sequences obtained in the present study were compared to those of AIVs from the GeneBank (Figures 1–8).

Figure 1 shows the phylogenetic tree of the complete H gene, subtype H6, of representative recent AIV H6 isolates reported worldwide, including the previous two Israeli H6 isolates, which were not reported to the GeneBank. The H genes examined formed two phylogenetic clades. It seems that the H gene of the present isolate, A/turkey/Israel/09 (H6N1), differed from the H genes of the two previous Israeli isolates, as they belonged to different clades, although the differences in the nucleotide content were about 3.7-3.8%. It is notable that the H6 AIV, that shaped the clade to which the present isolate belonged, originated from waterbirds.

Figure 2 shows the phylogenetic tree of the N gene of N1 subtypes, based on the analysis of nucleotides 204 to 1149. The analysis includes representative AIV N1 genes compared on the respective gene fragment. While the compared sequences differed up to 31%, the present isolate, A/turkey/Israel/09 (H6N1), was closest to the N1 from A/mute swan/Aktau/06 (H5N1) and A/swan/Mangystau/06 (H5N1). It is notable to mention that the Hong Kong avian A/teal/Hong Kong/W312/97 (H6N1) and the human influenza virus A/Hong Kong/156/97 (H5N1) showed a very high nucleotide homology in the 6 AIV internal genes, and especially in the N1 gene sequence [14, 24]. That similarity might indicate a common precursor and that the present virus could become a potential source of novel pathogenic AIV strains. 

Figures 3, 4, 5, 6, 7, and 8 show the phylogenetic comparison of the A/turkey/Israel/09 (H6N1) isolate complete internal genes, NS, M, NP, PA, PB1 and PB2, respectively, to genes of representative AIVs. The main feature revealed from these phylogenetic trees was the close similarity between the former two H6N2 AIV isolates, A/duck/Israel/289/01 and A/mallard/Israel/320/01, as compared to the phylogenetic divergence of the present H6N1 isolate, A/turkey/Israel/09. The phylogenetic distances between the 2009 H6N1 isolate and the two previous H6N2 isolates were calculated. While the two previously described H6 isolates, A/duck/Israel/289/01 (H6N2) and A/mallard/Israel/320/01 (H6N2), were identical in their NS, M, NP, PA, PB1, and PB2 genes, the A/turkey/Israel/09 (H6N1) isolate differed from the former two isolates by 3%, 2.3%, 6%, 2.5%, 3.6%, and 5%, respectively.

In conclusion, the present paper describes a third H6 AIV isolate (H6N1) in Israel, which unlike the two previous H6N2 isolates that were obtained from ducks and mallard, was now detected in turkeys. This H6N1 LPAIV was shown to possess a low-pathogenic amino acid sequence at the cleavage site and had a low IVPI. Phylogenetic analysis of all genes suggested a different origin for the virus. A/turkey/Israel/09 (H6N1) was not highly transmissible among commercial flocks. That phenomenon was supported by two observations; (a) the absence of clinically-affected cases in adjacent poultry houses and (b) the detection of only three cases of AIV H6 in Israel.

## References

[1] Capua I, Alexander DJ (2006). The challenge of avian influenza to the veterinary community. *Avian Pathology*.

[2] Wright PF, Webster RG, Knipe D, Howley P (2001). Ortomyxoviruses. *Fields, Virology*.

[3] Reid AH, Taubenberger JK, Fanning TG (2004). Evidence of an absence: the genetic origins of the 1918 pandemic influenza virus. *Nature Reviews Microbiology*.

[4] Taubenberger JK, Reid AH, Lourens RM, Wang R, Jin G, Fanning TG (2005). Characterization of the 1918 influenza virus polymerase genes. *Nature*.

[5] Webster RG, Bean WJ, Gorman OT, Chambers TM, Kawaoka Y (1992). Evolution and ecology of influenza A viruses. *Microbiological Reviews*.

[6] Cheung CL, Vijaykrishna D, Smith GJD (2007). Establishment of influenza A virus (H6N1) in minor poultry species in southern China. *Journal of Virology*.

[7] Shortridge KF (1992). Pandemic influenza: a zoonosis?. *Seminars in Respiratory Infections*.

[8] Munster VJ, Baas C, Lexmond P (2007). Spatial, temporal, and species variation in prevalence of influenza A viruses in wild migratory birds.. *PLoS Pathogens*.

[9] Spackman E, Stallknecht DE, Slemons RD (2005). Phylogenetic analyses of type A influenza genes in natural reservoir species in North America reveals genetic variation. *Virus Research*.

[10] Jackwood MW, Suarez DL, Hilt D (2010). Biologic characterization of chicken-derived H6N2 low pathogenic avian influenza viruses in chickens and ducks. *Avian diseases*.

[11] Abolnik C, Bisschop S, Gerdes T, Olivier A, Horner R (2007). Outbreaks of avian influenza H6N2 viruses in chickens arose by a reassortment of H6N8 and H9N2 ostrich viruses. *Virus Genes*.

[12] Wang CW, Wang CH (2003). Experimental selection of virus derivatives with variations in virulence from a single low-pathogenicity H6N1 avian influenza virus field isolate. *Avian Diseases*.

[13] Woolcock PR, Suarez DL, Kuney D (2003). Low-pathogenicity avian influenza virus (H6N2) in chickens in California, 2000–02. *Avian Diseases*.

[14] Hoffmann E, Stech J, Leneva I (2000). Characterization of the influenza A virus gene pool in avian species in southern China: was H6N1 a derivative or a precursor of H5N1?. *Journal of Virology*.

[15] Golender N, Panshin A, Banet-Noach C (2008). Genetic characterization of avian influenza viruses isolated in Israel during 2000–2006. *Virus Genes*.

[16] Perk S, Golender N, Banet-Noach C (2009). Phylogenetic analysis of hemagglutinin, neuroaminidase and nucleoprotein genes of H9N2 avian influenza viruses isolated in Israel during the 2000–2005 epizootic. *Comparative Immunology, Microbiology and Infectious Diseases*.

[17] Swayne DE, Senne DA, Beard CW, Swayne DE, Glisson JR, Jackwood MW, Pearson JE, Read WM (1998). Influenza. *Isolation and Identification of Avian Pathogens*.

[18] Lee MS, Chang PC, Shien JH, Cheng MC, Shieh HK (2001). Identification and subtyping of avian influenza viruses by reverse transcription-PCR. *Journal of Virological Methods*.

[19] Slomka MJ, Coward VJ, Banks J (2007). Identification of sensitive and specific avian influenza polymerase chain reaction methods through blind ring trials organized in the European Union. *Avian Diseases*.

[20] Wright KE, Wilson GAR, Novosad D, Dimock C, Tan D, Weber JM (1995). Typing and subtyping of influenza viruses in clinical samples by PCR. *Journal of Clinical Microbiology*.

[21] Office International des Epizooties, World organization for Animal Health (2001). Highly pathogenic avian influenza (fowl plague). *Manual of Standards for Diagnostic Tests and Vaccines*.

[22] Klenk HD, Matrosovich MN, Stech J (2008). Cleavage activation of the influenza virus hemagglutinin and its role in pathogenesis. *Avian Influenza*.

[23] Steinhauer DA (1999). Role of hemagglutinin cleavage for the pathogenicity of influenza virus. *Virology*.

[24] Chin PS, Hoffmann E, Webby R (2002). Molecular evolution of H6 influenza viruses from poultry in southeastern China: prevalence of H6N1 influenza viruses possessing seven A/Hong Kong/156/97 (H5N1)-like genes in poultry. *Journal of Virology*.

